# From Protocol to Practice: Enhancing Fidelity, Supporting Adaptation, and Advancing Science

**DOI:** 10.1093/geroni/igaf122.3776

**Published:** 2025-12-31

**Authors:** Claire Regan

**Affiliations:** University of Pennsylvania, Philadelphia, Pennsylvania, United States

## Abstract

As the global population ages, ensuring that evidence-based interventions (EBIs) are effectively translated into real-world settings is critical to meeting the complex and evolving needs of older adults. This population often experiences fragmented care and is particularly vulnerable to poor outcomes. Effective translation of EBIs hinges on balancing fidelity, the extent to which an intervention is delivered as originally intended, with the necessary adaptations required for diverse, real-world settings. Rigid adherence to fidelity can undermine local applicability and contextual relevance, but divergence from fidelity may compromise theoretical integrity and patient outcomes. This work explores the importance of and barriers to measuring and reporting both fidelity and adaptation, with a focus on their implications for successful implementation and scalability. Drawing on implementation science frameworks and using the Transitional Care Model (TCM) as a case study, we examine the interplay between fidelity, adaptation, and context. Emphasis is placed on creating practical tools, fostering bidirectional learning, and establishing fidelity standards in intervention protocols. The work concludes with recommendations aimed at enhancing training, reporting, and collaboration to support equitable and effective implementation of EBIs across diverse settings in meaningful ways.

